# Influence of Crystallinity and Energetics on Charge Separation in Polymer–Inorganic Nanocomposite Films for Solar Cells

**DOI:** 10.1038/srep01531

**Published:** 2013-03-25

**Authors:** Neha Bansal, Luke X. Reynolds, Andrew MacLachlan, Thierry Lutz, Raja Shahid Ashraf, Weimin Zhang, Christian B. Nielsen, Iain McCulloch, Dylan G. Rebois, Thomas Kirchartz, Michael S. Hill, Kieran C. Molloy, Jenny Nelson, Saif A. Haque

**Affiliations:** 1 Centre for Plastic Electronics and Department of Chemistry, Imperial College London, South Kensington Campus, Exhibition Road, SW7 2AZ, U.K.; 2 Centre for Plastic Electronics and Department of Physics, Imperial College London, South Kensington Campus, Exhibition Road, SW7 2AZ, U.K.; 3 Department of Chemistry, University of Bath, Claverton Down, Bath BA2 7AY, U.K

## Abstract

The dissociation of photogenerated excitons and the subsequent spatial separation of the charges are of crucial importance to the design of efficient donor-acceptor heterojunction solar cells. While huge progress has been made in understanding charge generation at all-organic junctions, the process in hybrid organic:inorganic systems has barely been addressed. Here, we explore the influence of energetic driving force and local crystallinity on the efficiency of charge pair generation at hybrid organic:inorganic semiconductor heterojunctions. We use x-ray diffraction, photoluminescence quenching, transient absorption spectroscopy, photovoltaic device and electroluminescence measurements to demonstrate that the dissociation of photogenerated polaron pairs at hybrid heterojunctions is assisted by the presence of crystalline electron acceptor domains. We propose that such domains encourage delocalization of the geminate pair state. The present findings suggest that the requirement for a large driving energy for charge separation is relaxed when a more crystalline electron acceptor is used.

Solution processable organic and hybrid organic:inorganic semiconductor nanocomposites are attracting intense interest for the development of low-cost, scalable and robust photovoltaic technologies^1^,^2^,^3^,^4^,^5^,^6^. Whilst power conversion efficiency (PCE) values in the region of 10% that are comparable with amorphous silicon have been reported^1^,^3^,^7^, the limiting available PCE – and consequently, the scale of application – will depend upon the factors controlling charge pair separation and is not as yet understood. It is commonly considered that the generation of free charges at a donor:acceptor heterojunction results from the dissociation of a photogenerated exciton to generate a bound complex with charge transfer (CT) character, and the separation of this CT complex into fully dissociated charge carriers (Figures 1a & 1b)^8^,^9^,^10^. Efficient charge generation requires that the separation process competes successfully with recombination of the bound CT complex and with energy transfer to singlet or triplet excitons of either material. There is some evidence that charge separation is assisted by an excess free energy^11^ and by the existence of large or ordered domains on either side of the heterojunction that enable charge delocalisation^12^. Both the energy dependence and the domain size dependence of charge separation yield could be explained by the presence of a series of CT states of different energy and spatial extent. Moreover, the dissociation of an energetic exciton to generate a higher lying CT state could then lead to greater spatial delocalization of the charges and a correspondingly lower barrier to charge separation^13^,^14^,^15^. Similarly, larger domain size would enable greater delocalization of the CT state.

In order to determine the limiting efficiency of heterojunction solar cells we need to establish the relationship between the nominal interfacial driving energy (defined here as the difference between the LUMO of the donor and the LUMO or conduction band of the acceptor, Δ*E*_CS_), the degree of local ordering and the charge separation efficiency. This requires the use of suitable model systems. Previous studies have focussed on polymer:polymer and polymer:fullerene systems^11^,^12^,^13^,^16^ but understanding of these systems is limited by the interdependence of the ordering of the two materials and by the poor contrast and low atomic number and thus mean electron density of the components, which limits the quality of microstructural information. An alternative system is the hybrid polymer:inorganic heterojunction where a nanostructured inorganic phase acts as electron acceptor. Hybrid systems are interesting model systems because of the relative ease of determining and controlling microstructure but also because of the potential for efficient charge separation at low Δ*E*_CS_ through the high dielectric permittivity of the inorganic phase and correspondingly low Coulombic binding energy. For example, one recent study indicated charge generation could result from triplet exciton dissociation at an organic lead sulfide interface at a Δ*E*_CS_ of only 0.05 eV^17^. Previously, hybrid materials were limited by the difficulty of fabricating heterojunctions that offered both close interpenetration of phases and high electronic connectivity within the inorganic phase. Recently, a precursor based fabrication protocol has been introduced where a xanthate precursor is co-processed with the organic phase and then converted into an interconnected sulfide network through a low temperature thermal conversion with the release of gaseous reaction products^18^,^19^. The resulting microstructure can be controlled through process conditions (composition ratio, temperature, additives)^20^,^21^. In particular, the inorganic semiconductor crystal growth can be controlled through the addition of Lewis base amine solvents^22^,^23^.

In spite of the potential for energetically inexpensive charge separation, hybrid PV materials have seldom been studied in this context. In this paper, we investigate the relative influence of crystallinity and driving energy on charge generation in hybrid polymer:metal sulfide nanocomposite films, using a series of polymers of varying electron affinity to control Δ*E*_CS_, and a sacrificial amine additive to modulate the inorganic phase crystallinity. We use steady state photoluminescence (PL) and transient absorption spectroscopy (TAS) to show that the yield of photogenerated charges is correlated to the crystallinity of the inorganic acceptor phase, as ascertained by x-ray diffraction (XRD), and that such crystallinity reduces the need for a large Δ*E*_CS_. Our observations suggest that while the yield of photogenerated charges in hybrid inorganic-organic devices depends upon both driving energy and crystallinity, the presence of well-ordered crystalline inorganic domains enables efficient charge generation even at extremely low driving energies (Δ*E*_CS_ < 0.1 eV). We discuss the implications of our findings for the design of all-organic and hybrid DA heterojunction solar cells.

## Results

**Figure 1:**
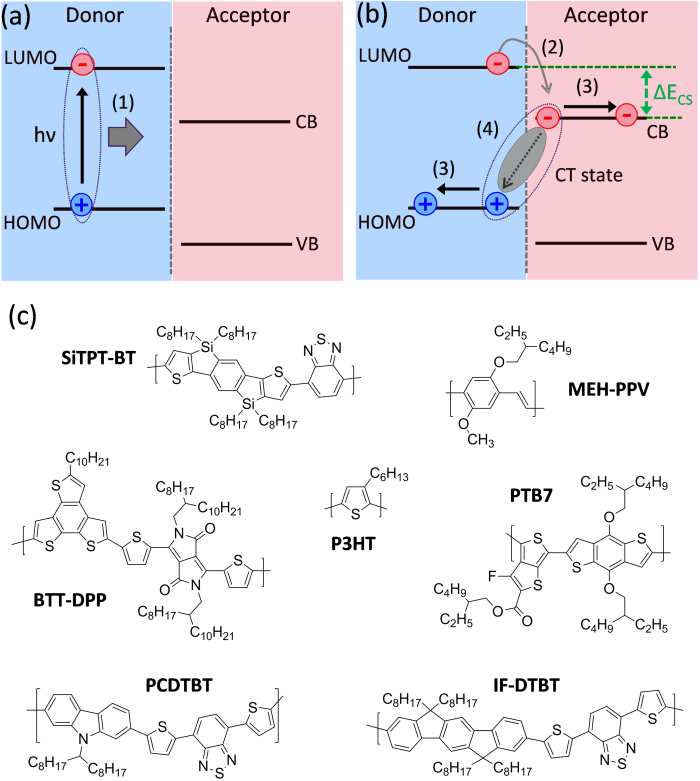
(a) and (b) show schematic diagrams of the charge separation process at donor-acceptor heterojunctions. Exciton generation via photon absorption is followed by exciton diffusion (process 1) to the D-A heterojunction. Charge separation (process 2) is followed by the formation of a CT state. Device performance depends critically on efficient dissociation of the polaron pair (process 3) avoiding geminate recombination (process 4). (c) shows the chemical structures of the polymers used in this study.

Polymer:cadmium sulfide (CdS) composite films were prepared through controlled *in-situ* thermal decomposition of a cadmium ethylxanthate pyridine adduct Cd(S_2_COEt)_2_.2C_5_H_5_N (CdPEX) in a polymer film as previously reported^21^. Figure 1c shows the chemical structures of the polymers investigated here. The ionization potentials, electron affinities, and singlet energies of the polymers along with Δ*E*_CS_ values for the CdS:polymer combinations are given in Table 1. Hybrid CdS:polymer films were fabricated by spin casting from a solution containing the metal xanthate precursor and the polymer followed by thermal annealing of the as-spun films at 160°C to generate the metal sulfide particle network within the polymer films. The crystallinity of the inorganic semiconductor phase (CdS) was tuned by the addition of a few volume percent of n-hexylamine to the polymer:precursor spin casting solution prior to film deposition and subsequent thermal annealing. This n-hexylamine is believed to leave the film during spin coating or subsequent thermal annealing as no change in the nitrogen profile of the films with and without n-hexylamine is observed using time of flight secondary ion mass spectroscopy (ToF-SIMS), see SI Figure S1, thereby confirming the absence of hexylamine in the final CdS:polymer nanocomposite film.

**Figure 2:**
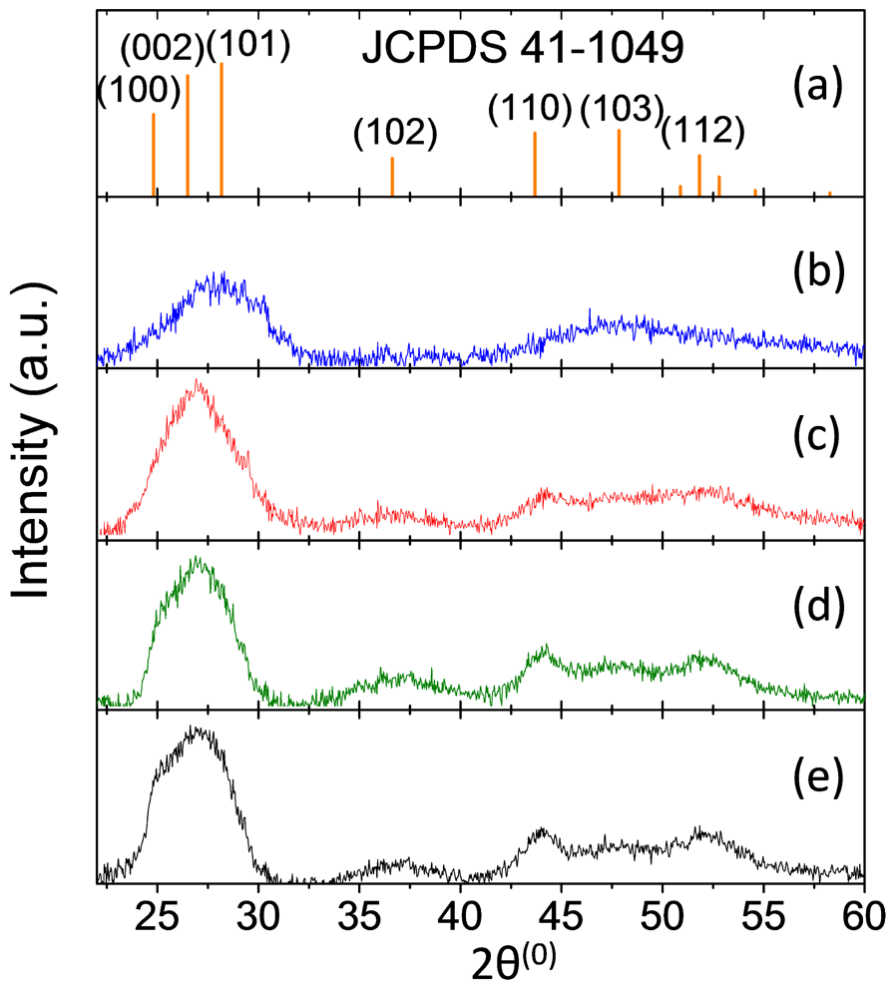
(a) Line spectrum for the CdS wurtzite hexagonal structure denoted JCPDS 41-1049. (b)–(e) x-ray diffraction data for the CdS:polymer samples (here PCDTBT) as a function of hexylamine concentration in the film-forming solution: (b) 0%, (c) 0.25%, (d) 1%, (e) 1.5% wt./vol.

**Figure 3:**
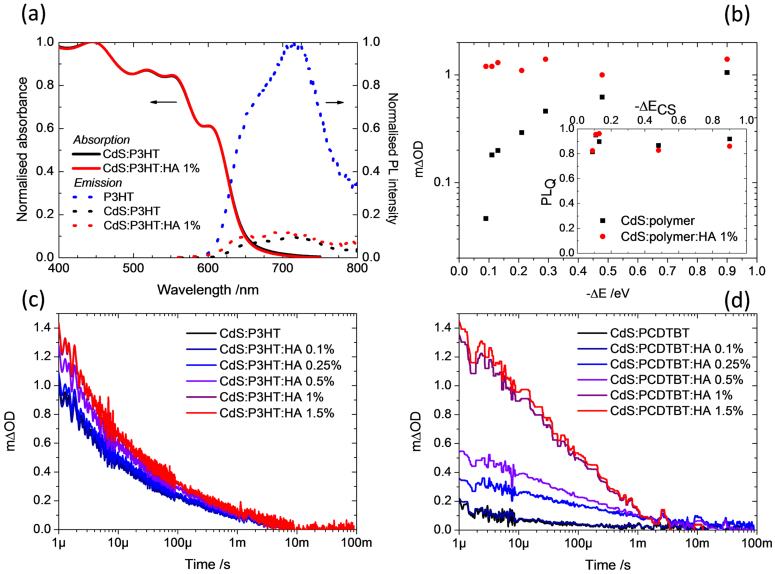
(a) Absorption and fluorescence spectra P3HT and P3HT:CdS in the presence and absence of 1% n-hexylamine (b) magnitude of the transient absorption signal (at 1 μs) as a function of driving energy for a series of CdS:polymer films in absence (black squares) and presence (red circles) of 1% n-hexylamine processing additive in the film-forming solution. Figure 3(b) inset shows the analogous PL quenching efficiency data for a series of CdS:polymer films in absence (black squares) and presence (red circles) of 1% hexylamine processing additive in the film-forming solution. (c) transient absorption kinetics for CdS:P3HT films as a function of n-hexylamine concentration in the film-forming solution. (d) transient absorption kinetics for CdS:PCDTBT films as a function of n-hexylamine concentration in the film-forming solution.

We first investigate the influence of the n-hexylamine additive upon the degree of crystallinity of the resultant CdS phase in our hybrid films. To this end, n-hexylamine was added to a chlorobenzene solution containing CdPEX and the polymer poly[N-9′-heptadecanyl-2,7-carbazole-alt-5,5-(4′,7′-di-2-thienyl-2′,1′,3′-benzothiadiazole)] (PCDTBT). The resulting solution was then spun cast onto a glass substrate and thermally annealed at 160°C under nitrogen for 1 hour. Figure 2 shows the XRD traces for the PCDTBT:CdS thin films as a function of n-hexylamine concentration in the spin coating solution. We note that the data shown in Figure 2 illustrates the XRD patterns of CdS after subtraction of the polymer and the glass contributions. The XRD patterns of the CdS samples are consistent with a wurtzite hexagonal phase structure (JCPDS 41-1049). The four peaks with 2*θ* values of 51.8, 47.8, 43.6 and 36.6 correspond to the (112), (103), (110) and the (102) planes of the hexagonal phase of CdS. It is apparent from the data presented in Figure 2 that the peaks become progressively sharper and better defined as the concentration of n-hexylamine is increased, an observation consistent with an increase in the CdS crystallite size. Furthermore, the appearance of the (100) diffraction peak at 2*θ* = 24.8 upon addition of hexylamine in the spin coating solution further indicates an increase in crystallinity of CdS. However, it is pertinent to note that the appearance of broad peaks as observed here is consistent with nanometer-sized crystallites or with a broad range of crystallite sizes including a nanometer sized fraction. In the case of a monodisperse distribution of spherical nanoparticles, the nanoparticle diameter according to the Debye Scherrer equation would be 2.5, 2.8, 3.5 and 3.7 nm for 0.25%, 0.5%, 1% and 1.5% wt./vol. n-hexylamine concentrations respectively (see SI Figure S2). For the case of no hexylamine, the corresponding diameter is in the range 1–2 nm. XRD studies on poly[3-hexylthiophene] (P3HT):CdS films in the absence and presence of n-hexylamine revealed a similar increase in the crystal diameter of the CdS, see SI Figure S2. Note that the absence of any evidence for quantum confinement in the absorption spectra (Figure 3(a) below) indicate that these coherence lengths are indicative of improved crystalline quality but not of actual nanocrystal sizes.

Figures 3(a) and SI Figure S3 show the steady state absorption characteristics of the CdS:polymer blends and pristine polymers investigated in this study. Figures 3(a) shows the normalized UV-vis absorption spectra of the CdS:P3HT films in the absence and presence (1% vol.) of the n-hexylamine additive in the spin coating solution. Both spectra exhibit the characteristic absorption of the respective polymer along with a shoulder at ~450 nm which we attribute to the absorption edge of the CdS phase as previously^18^. Furthermore, it is apparent that the addition of the n-hexylamine results in little or no change in the absorption characteristics of the blend films. This absence of any shift in absorption edge due to quantum size effects confirms that the coherence lengths obtained from XRD are indicate of CdS crystal quality rather than typical nanoparticle size. PL measurements were performed to evaluate the efficiency of exciton quenching at the CdS:polymer heterojunctions; PL quenching is a necessary, but not sufficient, condition for charge generation. Typical PL data obtained for CdS:P3HT are shown in Figure 3(a) as well as those for the corresponding pristine polymer. It is clear from the data presented here that the polymer emission is strongly quenched (> 85%) for all CdS:polymer blends studied. Figure 3(b) (inset) illustrates the PL quenching efficiency (PL_Q_) as a function of nominal driving energy (Δ*E*_CS_) for CdS:IF-DTBT, CdS:PCDTBT, CdS:PTB7, CdS:MEH-PPV and CdS:P3HT blend films in the absence and presence of 1% n-hexylamine in the spin coating solution. We note that the magnitude of the PL quenching is largely unaffected by the addition of n-hexylamine; high PL_Q_ values were observed in both cases (0% versus 1% n-hexylamine concentrations). Thus the PL quenching shows negligible dependence upon either driving force or crystallinity.

To obtain direct evidence for charge generation, TAS measurements were performed. Experimental details of our transient absorption spectrometer are given in the SI Figure S4. Pulsed laser excitation of the CdS:polymer films resulted in the appearance of transient absorption bands consistent with polaron formation (see SI Figure S4). The charge recombination dynamics were determined by monitoring the decay of the photoinduced polaron band of the donor polymer; control measurements performed in aerobic and anaerobic conditions indicate that the data presented in Figure 3 are due to photogenerated polarons rather than triplets (see SI Figure S5). Typical kinetic data for five of the CdS:polymer blends are shown in the SI, Figure S4. The kinetic traces follow the recombination of the photogenerated holes with electrons in the CdS. All samples were excited at a pump wavelength of 567 nm and power of 21 ± 2 μJcm^−2^, and the amplitude of the signal (mΔOD) is corrected for the ground state absorbance at the pump wavelength. Under these conditions mΔOD is proportional to the density of photogenerated transient species and the polaron extinction coefficient of the excited state. It is noteworthy that the extinction coefficients of the oxidized polymers IF-DTBT and P3HT (either end of the Δ*E*_CS_ scale) have previously been reported to be similar in magnitude (~20,000 and ~26,000 M^−1^ cm^−1^ for P3HT and IF-DTBT respectively^24^,^25^). Thus, the amplitude of the polaron absorbance, corrected for ground state absorbance, can be interpreted as a measure of charge generation efficiency. In the case of the CdS:polymer films which do not contain hexylamine (less crystalline CdS), the transient absorbance at 1 μs is plotted against the nominal driving energy for charge separation in Figure 3(b). Transient absorbance at 1 μs has previously been used to ascertain the charge generation yield in comparative studies, e.g. Refs 11, 16 and 26. For this series, a strong dependence of ΔOD upon Δ*E*_CS_ is observed: reducing Δ*E*_CS_ by 0.8 eV results in approximately a two orders of magnitude reduction in the polaron absorbance. Given that the exciton dissociation efficiency, monitored through PL quenching, is insensitive to Δ*E*_CS_ this observation is consistent with either the geminate recombination of bound charge pairs prior to separation or the failure of exciton dissociation to generate charges efficiently. The trapping of charge pairs in CT states has been observed previously in polymer:fullerene systems^26^ but only rarely suggested in inorganic-organic blends^27^. The increase in the yield of long-lived charges as a function of increasing Δ*E*_CS_ appears to be consistent with more efficient dissociation of the charge transfer state into free carriers when the driving force is higher, thereby avoiding parasitic geminate recombination losses^26^.

Modulation of the crystallinity of the inorganic CdS phase using n-hexylamine results in some remarkable changes in the yield of photogenerated charges as ascertained by TAS; the magnitude of the TAS yield increases with increasing crystallinity of the inorganic phase, as shown in Figures 3(c) and 3(d). We note that the transient absorption spectra in each case are unchanged by the addition of hexylamine, indicating the same photoexcited species are observed. Whilst this effect is apparent for all of the polymers examined in this study, the relative increase in the yield of photogenerated charges is greatest when Δ*E*_CS_ is small and the efficiency of charge generation without n-hexylamine is correspondingly small. For example, in the case of CdS:PCDTBT (Δ*E*_CS_ ≈ 0.1 eV) the transient absorption signal magnitude at 1 μs increases from 0.18 mΔOD to 1.3 mΔOD (~7-fold increase) upon addition of 1% n-hexylamine (Fig. 3(d)). In contrast, for CdS:P3HT (Δ*E*_CS_ ≈ 0.9 eV) a relatively small 1.5-fold increase in the yield of long-lived charges is observed (Fig. 3(c)). A plot of the transient absorption signal amplitude at 1 μs versus Δ*E*_CS_ is shown in Figure 3(b) for blends processed with 1% n-hexylamine. From this plot it is clear that the yield of charge photogeneration is essentially independent of Δ*E*_CS_ for the series of CdS:polymer films formed in the presence of 1% n-hexylamine, in contrast to the case without hexylamine. The strong dependence of polaron yield as a function of Δ*E*_CS_ for the n-hexylamine free CdS:polymer blends is consistent with the need for additional free energy to enable photoinduced polaron pairs to overcome their Coloumb attraction. Similar behaviour has been observed for polymer-polymer and polymer-fullerene heterojunctions^11^. In those cases, the influence of driving energy upon charge separation yield has been rationalized in terms of the excess energy of ‘hot’ charge transfer states that may be generated by higher energy photoexcitations. One proposal is that the hot charge transfer state loses its excess energy through vibrational relaxation leading to a greater spatial separation between the electron and hole possibly assisted by disorder in the electronic states^28^. Another is that the charge transfer exciton is generated in an electronically excited CT state in which the charges are less tightly bound than in the lowest CT state^10^,^13^,^29^. In either case, the excess energy increases the spatial separation of charges and reduces the barrier to charge separation. Our observation of a strong dependence of charge separation yield upon Δ*E*_CS_ in blends with a less crystalline inorganic phase is consistent with the notion of charge separation facilitated by excess energy of the photoexcited state. However, the effect of increased Δ*E*_CS_ is significantly less pronounced in blends with more crystalline CdS: in these cases, efficient charge separation is achieved with nominal driving energies as small as 0.1 eV.

## Discussion

The behaviour observed in this series of experiments may result from an influence of n-hexylamine on the interfacial energetics, increasing the actual Δ*E*_CS_ above the nominal value, or it may result from increased crystallinity of the inorganic component upon n-hexylamine addition with concomitant increased delocalisation of the CT states. A third possible explanation could be an increase in polymer or CdS domain size upon hexylamine addition, leading to the availability of more delocalised states or more escape routes from the interface^12^. We now address each of these possible explanations in more detail.

To rule out the possibility of different blend microstructure upon n-hexylamine addition, we obtained TEM images of CdS:P3HT films as a function of n-hexylamine concentration (SI Figure S6). These data show that n-hexylamine addition results in little or no change in the blend film microstructure. Furthermore, almost identical microstructures can be seen for the CdS:IF-DTBT, CdS:PCDTBT and CdS:P3HT samples, with and without hexylamine, (Fig. S6b and S6c) indicating that the differences in charge generation yield between polymers (Figure 2) cannot be attributed to variations in blend microstructure. This microscopic evidence is corroborated by the PL data in Figure 3, demonstrating similar PL quenching for all blend films.

**Figure 4:**
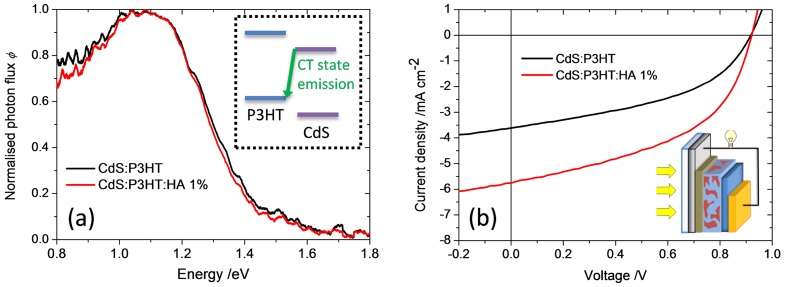
(a) Comparison of the normalized electroluminescence intensity for two CdS:P3HT solar cells with (1%) and without hexylamine measured at an injection current of *I* = 2 mA (corresponding to *J* ≈ 44 mAcm^−2^ at a pixel size of 0.045 cm^2^). The electroluminescence spectra show charge transfer emission as the emission of the blend is much lower in energy than both the emission from the P3HT (around 1.75 eV) and the CdS. The inset graphic displays the energetic origin of the CT state emission. (b) Current-voltage characteristics of CdS:P3HT solar cells with (1%) and without the n-hexylamine processing additive. Devices were prepared in the ‘inverted’ glass/ITO/TiO_x_/active layer/PEDOT:PSS/Au configuration as shown in the inset diagram and detailed in the SI.

To address whether the improved charge separation originates from a change in interfacial energetics upon processing with hexylamine, we use electroluminescence (EL) spectroscopy to estimate the energy of the charge transfer state at the polymer:CdS interface. EL has previously been used to determine the energy of emissive charge transfer states in polymer-fullerene solar cells^30^,^31^,^32^,^33^,^34^. For CdS:P3HT samples processed with and without hexylamine, see Figure 4(a) and S8, we find a broad emission peak at a photon energy *E* ≈ 1.1 eV, which is substantially below the absorption edge of both P3HT and CdS and the luminescence peak of P3HT. No electroluminescence was observed from pure CdS devices, indicating that the emission does not originate from defect states in the CdS. Thus, this measurement provides direct evidence for the existence of interfacial CT states at inorganic-organic heterojunctions, which has so far been rarely reported in literature^27^,^35^. The fact that the EL spectrum is insensitive to the use of n-hexylamine indicates that the additive does not change the interfacial energy line-up and therefore does not influence the driving energy for charge separation, Δ*E*_CS_.

**Figure 5:**
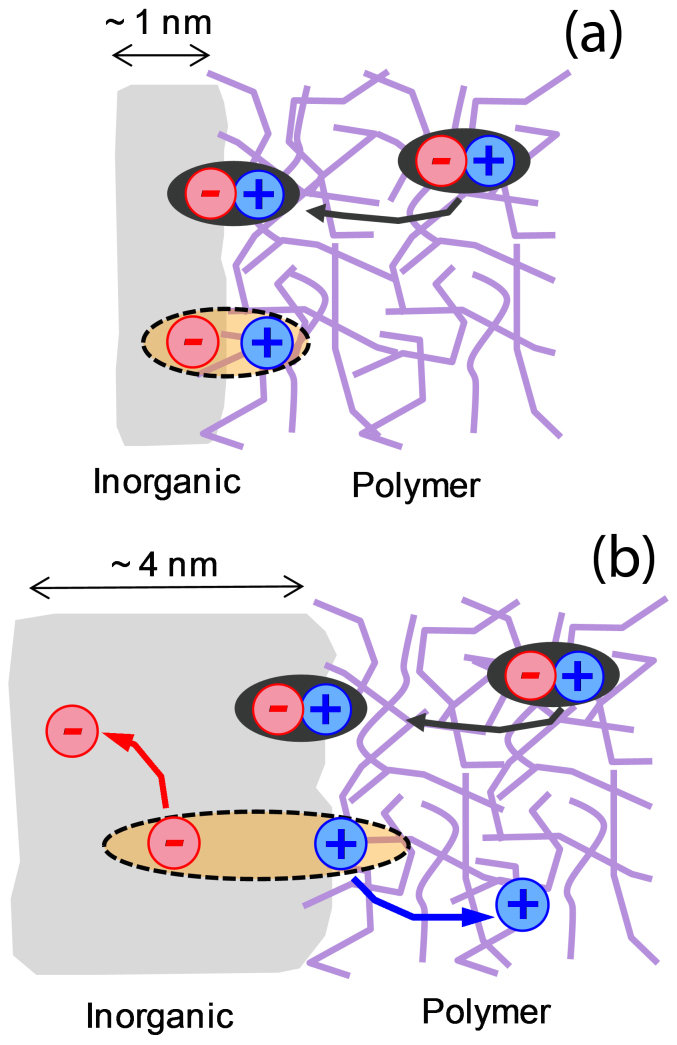
In the case of low Δ*E*_CS_ and small CdS crystallites (a) the charges remain bound leading to enhanced geminate recombination losses and therefore a lower yield of charge photogeneration. At low driving energies (Δ*E*_CS_) the generation of polarons is facilitated by larger CdS crystallites (b).

Finally we address the effect of inorganic component crystallinity on charge separation. The XRD data presented in Fig. 2 show that the degree of crystallinity of the CdS phase increases on n-hexylamine addition. If the XRD peak widths could be interpreted in terms of a monodisperse crystallite size then we should observe a change in microstructure and a progressive red shift of the CdS absorption edge as hexylamine is added. However, the TEM images of blend films (SI Figure S7) and the absorption spectra of CdS films and blends (SI Figure S3) show that the film microstructure and absorption spectra, respectively, appear unchanged by hexylamine. However, the absorption edge of the CdS phase becomes sharper upon hexylamine addition in the case of CdS:polystyrene blends (SI Figure S3) indicating the removal of defect states. We therefore argue that the role of the hexylamine is to improve the quality and size of pure CdS crystallites that form within a heterogeneous amorphous–crystalline CdS phase. Such enlarged domains of high quality crystalline CdS domains can be expected to support charge transfer states in which the electron is more delocalized within the CdS, resulting in a lower Coulomb binding energy and correspondingly easier separation of the charges away from the interface. The fact that efficient charge separation occurs at driving energies as low as 0.1 eV when the CdS crystallinity is high enough suggests that the threshold for charge separation at hybrid interfaces is lower than at organic:organic interfaces, possibly on account of the higher dielectric permittivity. Within a simple picture of Coulombic binding, a Coulomb binding energy of 0.1 eV or less corresponds to a charge separation distance of 1.4 nm or more, within a medium with the relative dielectric permittivity (of 9–10)^36^ of CdS. When hexylamine is added, crystalline CdS domains of sufficient size are generated. In the case of organic junctions of relative dielectric permittivity of around 3, facile charge separation would require charge delocalization over larger distances, of approximately 5 nm in size or greater, and highly crystalline organic domains of such sizes are not readily formed. We therefore propose that in the case of an organic:crystalline inorganic junction the Coulomb barrier for charge separation is relatively low compared to organic heterojunctions, and can be overcome provided that crystalline inorganic domains large enough to allow charge delocalization within the CT state are present. In cases where the inorganic crystallites are insufficiently large, the Coulomb binding energy is increased in accordance with the reduced charge delocalization distance, and the available driving force now determines whether or not the charge pairs can separate efficiently. In the case of a large Δ*E*_CS_, the Coulomb binding energy due to electron trapping in a small crystallite may be overcome while in the case of a low Δ*E*_CS_ and small crystallites, the charges remain bound. Such a phenomenon is schematically depicted in Figure 5. This picture would explain the observed dependence of charge separation efficiency on both crystallinity and driving energy. On the basis of these observations, it is expected that the improved charge separation yields (as a result of increasing crystallinity) should result in increased current generation and therefore improved device performance. Such an improvement in photocurrent generation and device performance is clearly demonstrated for CdS:P3HT as illustrated in Figure 4(b).

In conclusion, the present findings suggest that the energy cost associated with separating excitons and bound polaron pairs can be reduced by the use of better ordered donor and acceptor domains. In the current case, it is likely that the more crystalline acceptor encourages charge delocalization, thereby easing the requirement of excess free energy and offering a potential design strategy to optimize charge separation and device performance. Although the advantage of domain crystallinity for charge separation has also been observed in organic junctions^16^, the binding energy in organic junctions of the same dimensions or degree of crystallinity is higher than in hybrids on account of the lower dielectric permittivity, and the cost of charge separation is greater. The results presented here indicate that, with controlled microstructure, hybrid systems may achieve charge separation at an energetic cost that is some few tenths of an eV lower than for typical organic heterojunctions.

## Methods

### Sample preparation and characterization

Hybrid CdS:polymer films were prepared from a chlorobenzene solution containing 65 mg/ml Cd(S_2_COEt)_2_.2C_5_H_5_N and 4 mg/ml of the polymer. Films were prepared by spin coating at 1000 rpm for 60 seconds. The resultant as-spun films were then annealed in a N_2_ atmosphere at 160°C for 60 minutes. To control the crystallinity of the CdS, samples were spin coated from a chlorobenzene solution containing Cd(S_2_COEt)_2_.2C_5_H_5_N and polymer (as above) containing the n-hexylamine additive. CdS:P3HT photovoltaic devices were fabricated by spin coating a hybrid film onto a dense CdS/TiO_2_ coated ITO substrate as previously reported^21^. The thickness of the resulting active layers was in the range 160–180 nm. Device fabrication was completed by spin coating PEDOT:PSS onto the resultant CdS:P3HT photoactive layer before deposition of ~150 nm of Ag as the top electrode. Blend samples for TEM were fabricated on water soluble sacrificial substrates (PEDOT:PSS) on glass and floated onto the surface of deionised water before being caught on 300 mesh copper grids for top-down imaging. TEM was performed using a JEOL 2000 MkII electron microscope operated at 200 kV. XRD measurements were performed with a PANalytical X'Pert Pro MRD diffractometer using Ni filtered Cu K-alpha radiation at 40 kV and 40 mA.

### Optical Spectroscopy

Steady state absorption and emission measurements were performed using a Perkin-Elmer Lambda 25 spectrophotometer and Horiba Jobin Yvon Fluorolog-2 respectively. Micro- to milli-second transient absorption spectroscopy was performed on films under a N_2_ environment and all data shown is scaled for the fraction of photons absorbed at the excitation wavelength. The samples were excited by a dye laser (Photon Technology International Inc. GL-301) pumped by a nitrogen laser (Photon Technology International Inc. GL-3300) to give a pulse width of 0.6 ns at 4 Hz. Excitation was at 567 nm at an energy of 21 ± 2 μJcm^−2^. The samples were probed using a halogen lamp (Bentham, IL1) with a stabilised power supply (Bentham, 605). The probe light was detected using a silicon or In_x_Ga_1−x_As photodiode and the signal subsequently amplified and passed through electronic band-pass filters to improve the signal to noise ratio. The probe wavelengths were 950 nm (SiIDT-BT), 1160 nm (BTT-DPP), 940 nm (IF-DTBT), 1200 nm (PTB7), 980 nm (PCDTBT and P3HT), and 940 nm (MEH-PPV).

### Current/voltage measurements

Current-voltage characteristics of the cells were measured using a 150 W Xenon lamp (ScienceTech SS150W solar simulator) equipped with an IR filter (Water Filter) and AM1.5 filter (ScienceTech).

### Electroluminescence measurements

Electroluminescence was measured using a Princeton Instruments Acton SP 2500 spectrograph combined with a liquid nitrogen-cooled InGaAs photodiode array (Acton OMAV:1024). The spectral intensity was corrected with the spectrum from a calibrated halogen lamp.

## Author Contributions

N.B. prepared samples, performed optical spectroscopy measurements, fabricated and characterized solar cells, performed XRD studies and analysed data. L.X.R. assisted with the experiments and analysed the data. A.M. performed the microscopy measurements and assisted with the synthesis of the inorganic compound. T.L. synthesized the inorganic compound and assisted with the XRD measurements. R.S.A., W.Z., C.B.N. and I.M. synthesized polymers. D.G.R. and T.K. performed the electroluminescence measurements. M.S.H. and K.M. assisted with inorganic materials synthesis and discussed data. J.N. designed, planned and guided the work. S.A.H. initiated the project and designed, planned and guided the work. S.A.H., J.N., L.X.R. and T.K. co-wrote the manuscript. All the authors reviewed the manuscript.

## Supplementary Material

Supplementary Information Supplementary info

## Figures and Tables

**Table 1:** Shows electron affinity (*EA*) of the polymer, ionization potential (*IP*) of the polymer and driving energy Δ*E*_CS_. Δ*E*_CS_ = *E*_S_ − [*IP*_polymer_ − *CB*_CdS_] where *E*_S_, *IP*_polymer_ and *CB*_CdS_ are the singlet energy of the polymer, ionization potential of the donor and conduction band energy of the CdS respectively. *CB*_CdS_ = −3.71 eV

Polymer	*−EA*/eV	*−IP*/eV	−*E*_S_/eV	−Δ*E*_CS_/eV
IF-DTBT	3.73	5.60	1.98	0.09
PCDTBT	3.60	5.50	1.90	0.11
SiIDT-BT	3.68	5.48	1.90	0.13
BTT-DPP	3.60	5.20	1.70	0.21
PTB7	3.31	5.15	1.73	0.29
MEH-PPV	3.00	5.07	1.84	0.48
P3HT	3.20	4.80	2.00	0.90
