# Supplementary material for: Influence of Crystallinity and Energetics on Charge Separation in Polymer–Inorganic Nanocomposite Films for Solar Cells
Source: Sci Rep. 2013 Mar 25;3:1531. doi: 10.1038/srep01531 (PMC3607122; doi:10.1038/srep01531)
Supplement: Supplementary Information — Supplementary info [file srep01531-s1.pdf]

Supplementary Information for "Influence of Crystallinity and Energetics on Charge Separation in Polymer-Inorganic Nanocomposite Films for Solar Cells"

Neha Bansal[1], Luke X. Reynolds[1], Andrew MacLachlan[1], Thierry Lutz[1], Raja Shahid Ashraf[1], Weimin Zhang[1], Christian B. Nielsen[1], Iain McCulloch[1], Thomas Kirchartz[2], Jenny Nelson[2], Mike S. Hill[3], Kieran Molloy[3] and Saif A. Haque[1]**

[1] Centre for Plastic Electronics and Department of Chemistry, Imperial College London, South Kensington Campus, Exhibition Road, SW7 2AZ, U.K.

[2] Centre for Plastic Electronics and Department of Physics, Imperial College London, South Kensington Campus, Exhibition Road, SW7 2AZ, U.K.

[3] Department of Chemistry, University of Bath, Claverton Down, Bath BA2 7AY, U.K.

Corresponding authors: s.a.haque@imperial.ac.uk and jenny.nelson@imperial.ac.uk

Content:

Figure S1: Time-of-flight secondary ionizaiton mass spectrometry (ToF-SIMS) technique and data

Figure S2: XRD data and analysis

Figure S3: Steady state absorption and emission characteristics

Figure S4: Transient absorption spectroscopy technique and polaron spectra / kinetics

Figure S5: Transient absorption data obtained in aerobic and anaerobic environments

Figure S6: Transmission electron microscopy

Figure S7: Polymer details

Figure S8: Electroluminescence data

Figure S1

Time-of-Flight Secondary Ionisation Mass Spectrometry (ToF-SIMS)

Technique

A pulsed beam of bismuth ions is directed to a sample surface. The ions liberate neutral and charged elemental and molecular species from the surface. The positive and negatively ions can be selectively extracted and detected using a Time-of-Flight system in which ions are separated according to their mass. A separate, rastered beam of caesium ions is used to remove material from the surface over a defined area. Analysis at the centre of the crater is carried out using the bismuth beam. The analysis/etch process is repeated roughly every second until the film has been penetrated and the glass substrate reached.

Experimental

The ION-TOF 5 Time of Flight (ToF) Secondary Ion Mass Spectrometry (SIMS) instrument was used to obtain compositional depth profile through the coatings. The analysis beam was Bi_3^+ and the sputter beam was 1 keV Cs^+ with a beam current of 74.3 ± 0.1 nA. The sputter beam was rastered over 300 x 300 micron area and the analysis beam scanned over a 70 x 70 µm area at the centre of the sputtered region. Caesium forms positive and negative ion clusters with neutral species that are sputtered from the surface. The signal level of CsM^+ clusters are less matrix dependent than from M^+ ions and are the ion species of choice, taking into account other factors such as peak intensity and peak overlaps.

Results

The addition of hexylamine to the film-forming solution has no visible effect on the nitrogen concentration (Figure S1).

Figure S1. Ratio of nitrogen to cadmium peak area ratio through the coatings for CdS:P3HT (black) and CdS:P3HT:HA 1% (red) films.

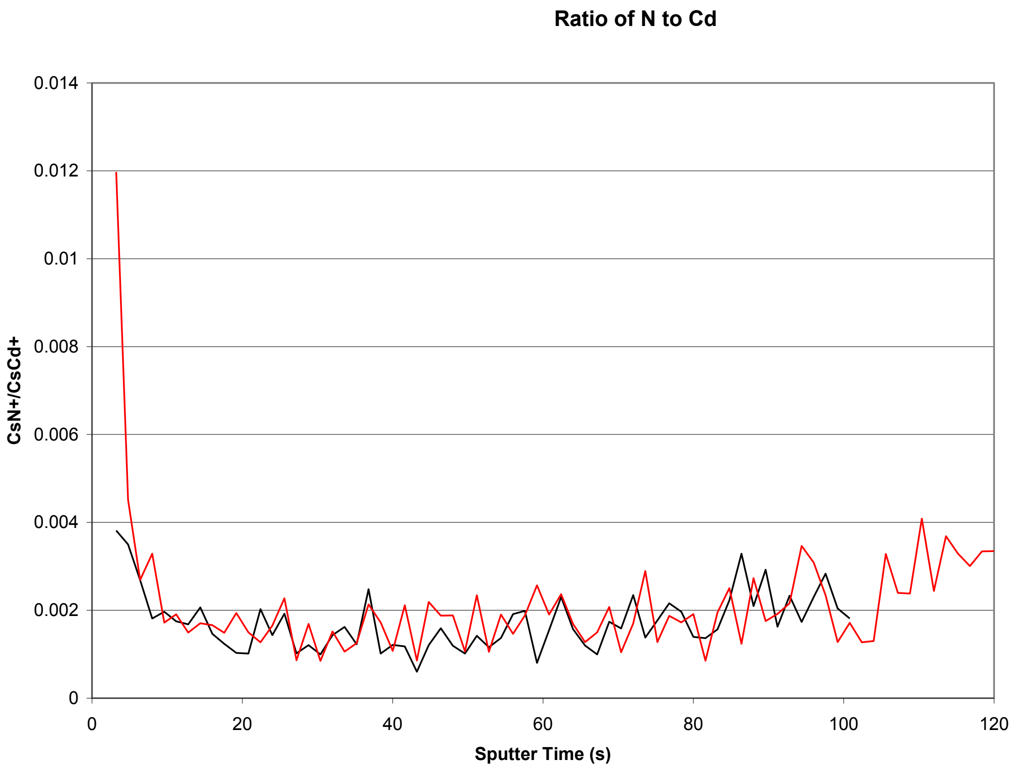

Figure S2

XRD data and analysis

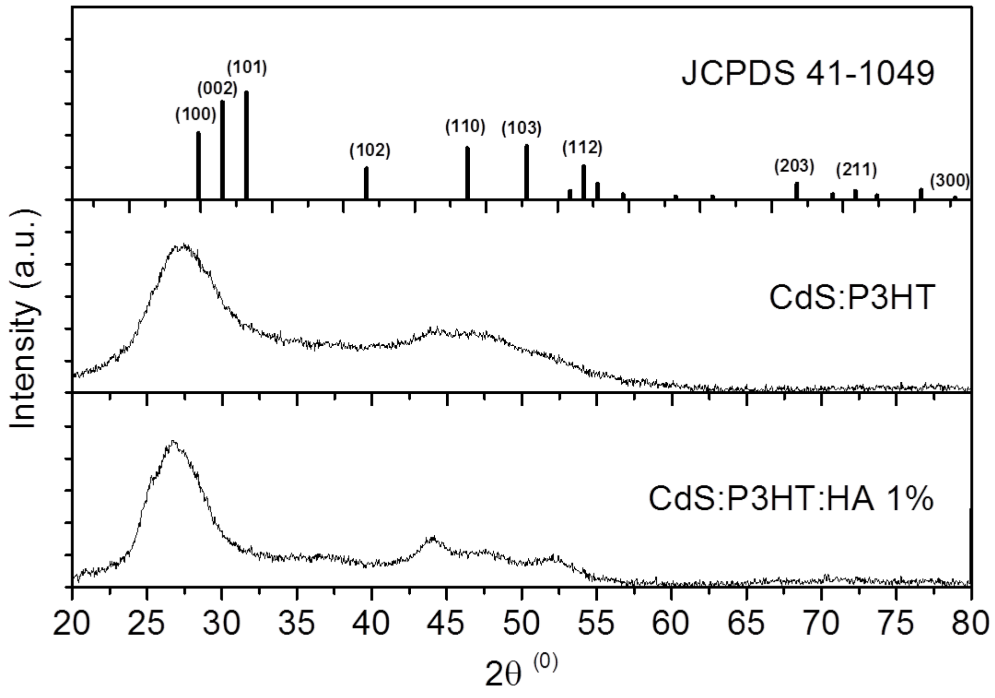

Figure S2. Line spectrum for the CdS wurtzite hexagonal structure denoted JCPDS 41-1049, and x-ray diffraction data for CdS:P3HT samples without and with 1% hexylamine.

The crystal diameter (L) of the CdS was estimated from the line broadening of the (110) diffraction peak using the Debye-Scherrer equation:

$L = K\lambda / \beta \cos\theta$

where K is the shape factor, λ is the wavelength of the x-ray radiation, β is the FWHM of the (110) peak and θ the diffraction angle. Assuming spherical crystallites (and equal size for all nanoparticles) (K = 0.9), the corresponding crystal diameters obtained in this way are ~1.0, 2.5, 2.8, 3.5 and 3.7 nm for 0%, 0.25%, 0.5%, 1% and 1.5% wt./vol. hexylamine concentrations respectively.

Figure S3

Steady state absorption and emission characteristics

Absorption:

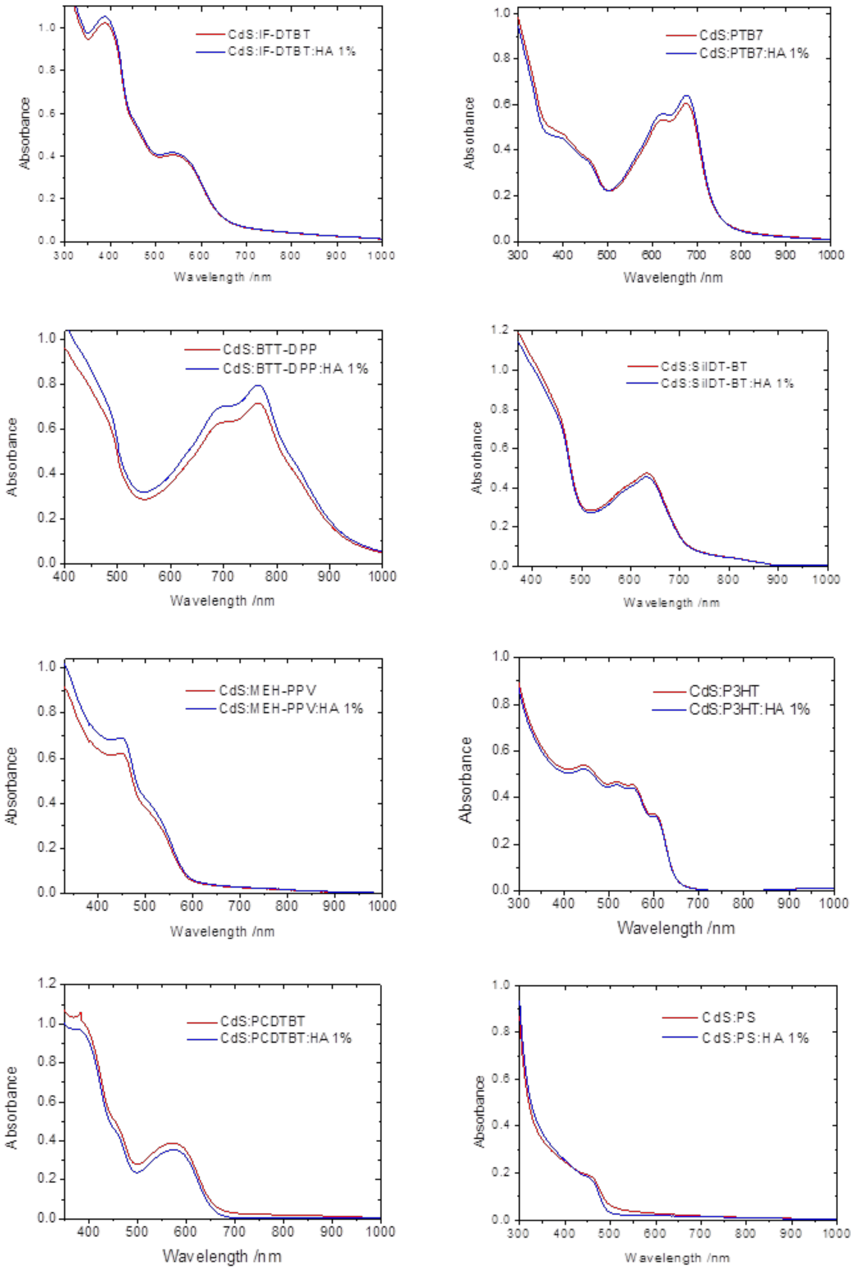

Figure S3a. Steady state absorption spectra of CdS:polymer films both without and with (1% wt./vol.) hexylamine processing additive in the film-forming solution. Seven of the polymers are detailed in the main text, with PS being polystyrene.

Emission:

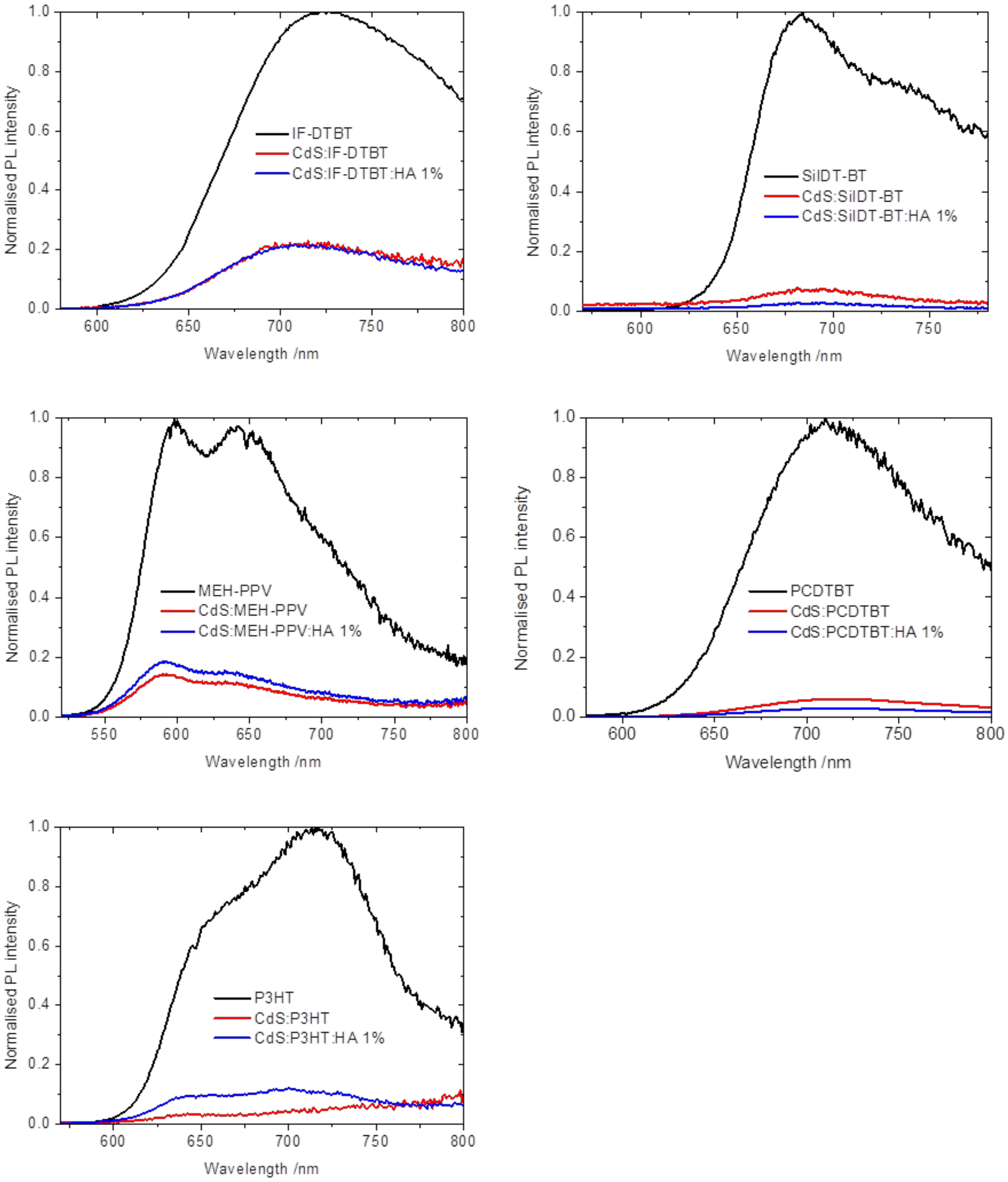

Figure S3b. Steady state emission spectra of pristine polymers, and CdS:polymer blend films both without and with (1% wt./vol.) hexylamine processing additive in the film-forming solution. The data is presented normalised to the pristine polymer PL intensity. Excitation wavelength for the samples were P3HT (555 nm), MEH-PPV (510 nm), PCDTBT (570 nm), IF-DTBT (570 nm), SiIDT-BT (550 nm).

Figure S4

Transient absorption spectroscopy technique and polaron spectra / kinetics

TAS experimental

Micro- to milli-second transient absorption spectroscopy was performed on films under a dynamic N_2 environment and all data shown is scaled for the fraction of photons absorbed at the excitation wavelength. The samples were excited by a dye laser (Photon Technology International Inc. GL-301) pumped by a nitrogen laser (Photon Technology International Inc. GL-3300) to give a pulse width of 0.6 ns at 4 Hz. Excitation was at 567 nm at an energy of 21 ± 2 μJcm^{-2}. The samples were probed using a quarts halogen lamp (Bentham, IL1) with a stabilised power supply (Bentham, 605). The probe wavelengths were 950 nm (SiIDT-BT), 1160 nm (BTT-DPP), 940 nm (IF-DTBT), 1200 nm (PTB7), 980 nm (PCDTBT and P3HT), and 940 nm (MEH-PPV). The probe light was detected using a silicon or $In_xGa_{1-x}As$ photodiode and the signal subsequently amplified and passed through electronic band-pass filters to improve the signal to noise ratio.

`

TAS spectra

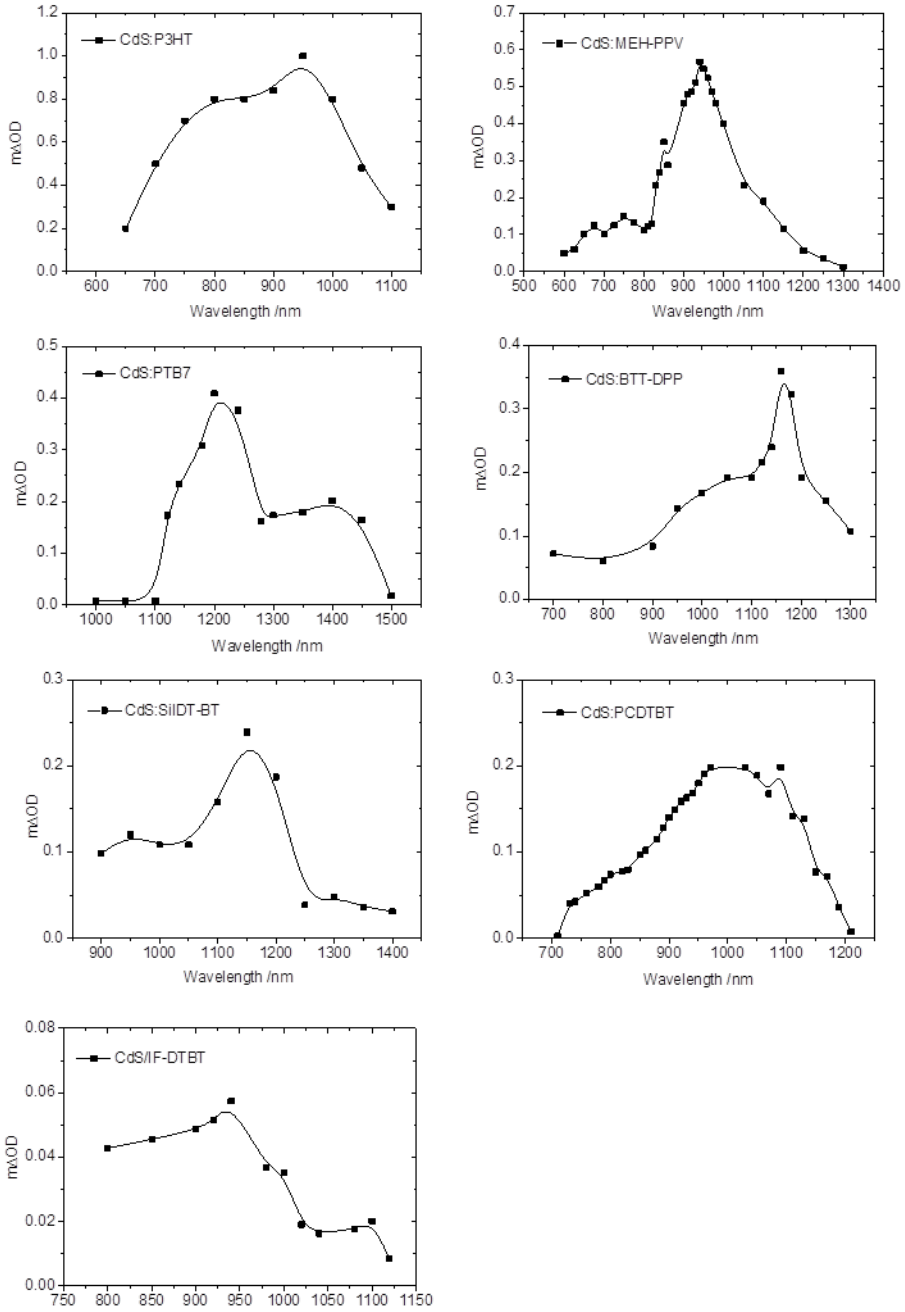

Figure S4a. Transient absorption spectra at 10 μs for the CdS:polymer blends used in this study. Excitation was at 567 nm at an energy of 21 ± 2 μJcm^{-2}.

Remaining TAS kinetics

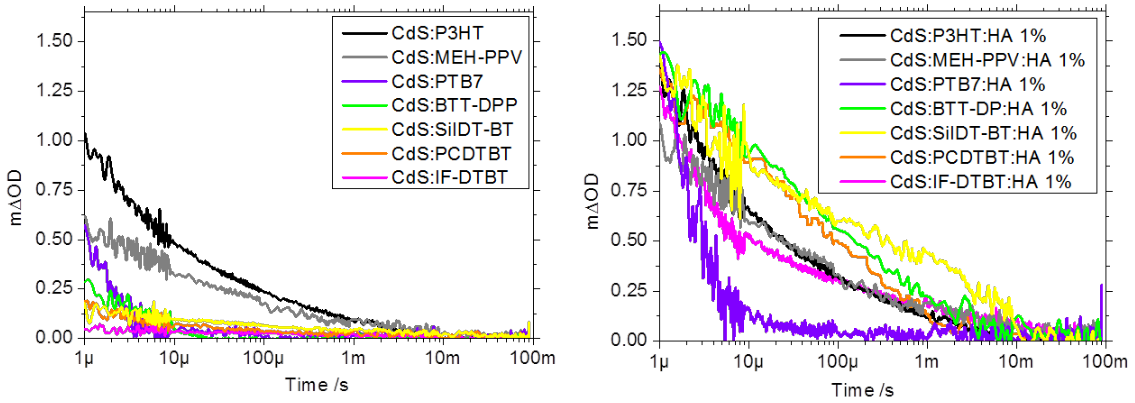

Figure S4b. Transient absorption kinetics at the polaron peak for CdS:polymer blends both without and with (1% wt./vol.) hexylamine processing additive in the film-forming solution. Excitation was at 567 nm at an energy of 21 ± 2 μJcm^{-2}. The probe wavelengths were 950 nm (SiIDT-BT), 1160 nm (BTT-DPP), 940 nm (IF-DTBT), 1200 nm (PTB7), 980 nm (PCDTBT and P3HT), and 940 nm (MEH-PPV).

Figure S5

Transient absorption data obtained in aerobic and anaerobic environments

Aerobic and Anaerobic TAS measurements

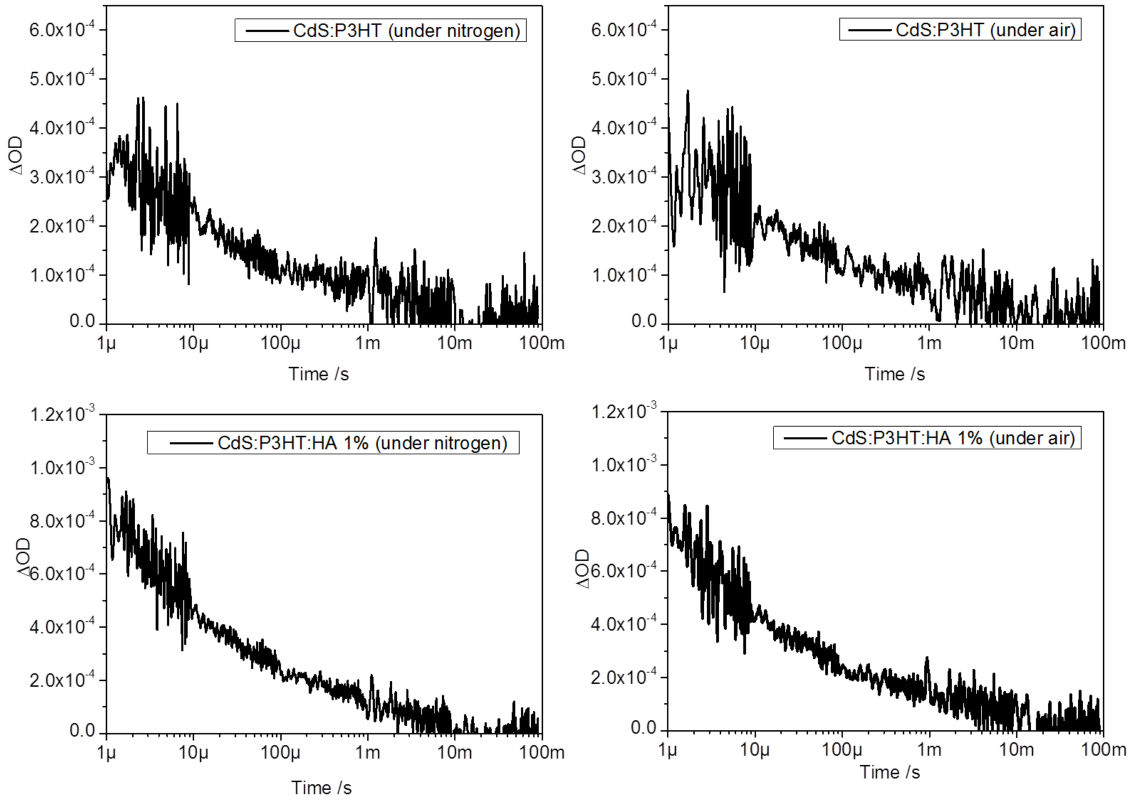

Figure S5a. Transient absorption kinetics at the polaron peak for CdS:P3HT blends both without and with (1% wt./vol.) hexylamine processing additive in the film-forming solution. Excitation was at 567 nm at an energy of 21 ± 2 μJcm^{-2}. The probe wavelength was 980 nm. The lack of change in signal when moving from a nitrogen to an air atmosphere confirms the transient absorption band is due to polarons and not triplets.

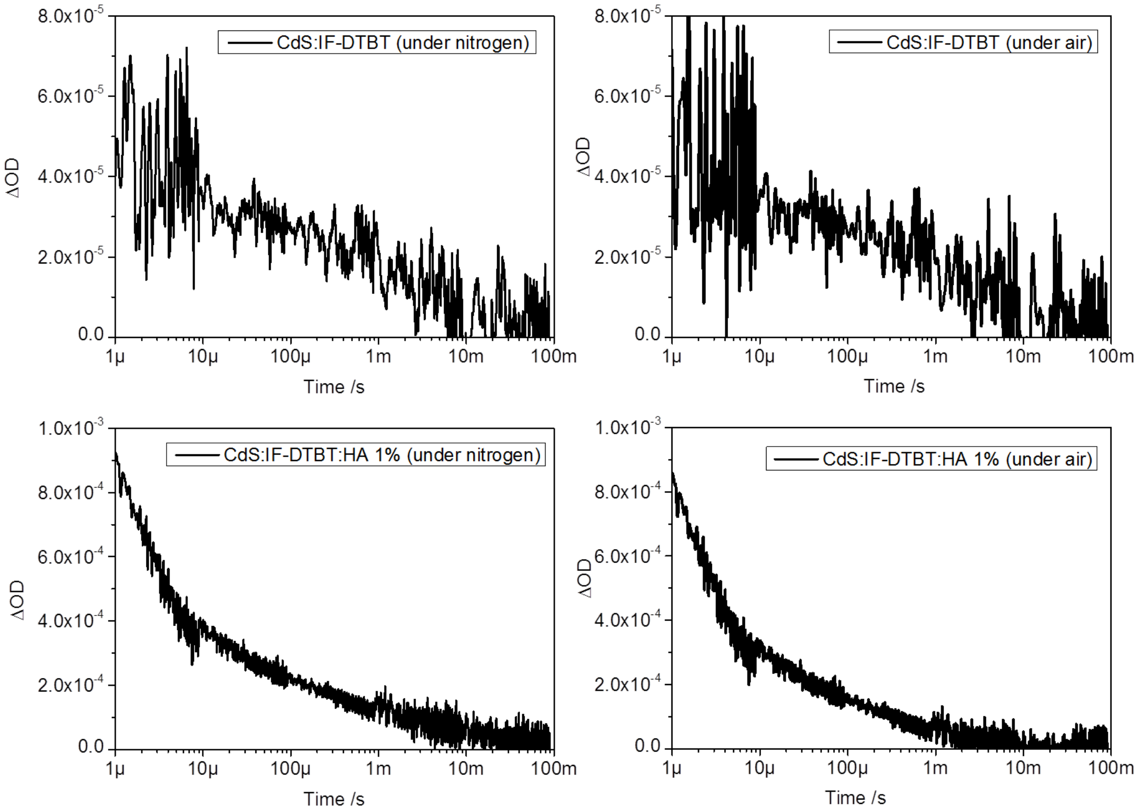

Figure S5b. Transient absorption kinetics at the polaron peak for CdS:IF-DTBT blends both without and with (1% wt./vol.) hexylamine processing additive in the film-forming solution. Excitation was at 567 nm at an energy of 21 ± 2 μJcm^{-2}. The probe wavelength was 940 nm. The lack of change in signal when moving from a nitrogen to an air atmosphere confirms the transient absorption band is due to polarons and not triplets.

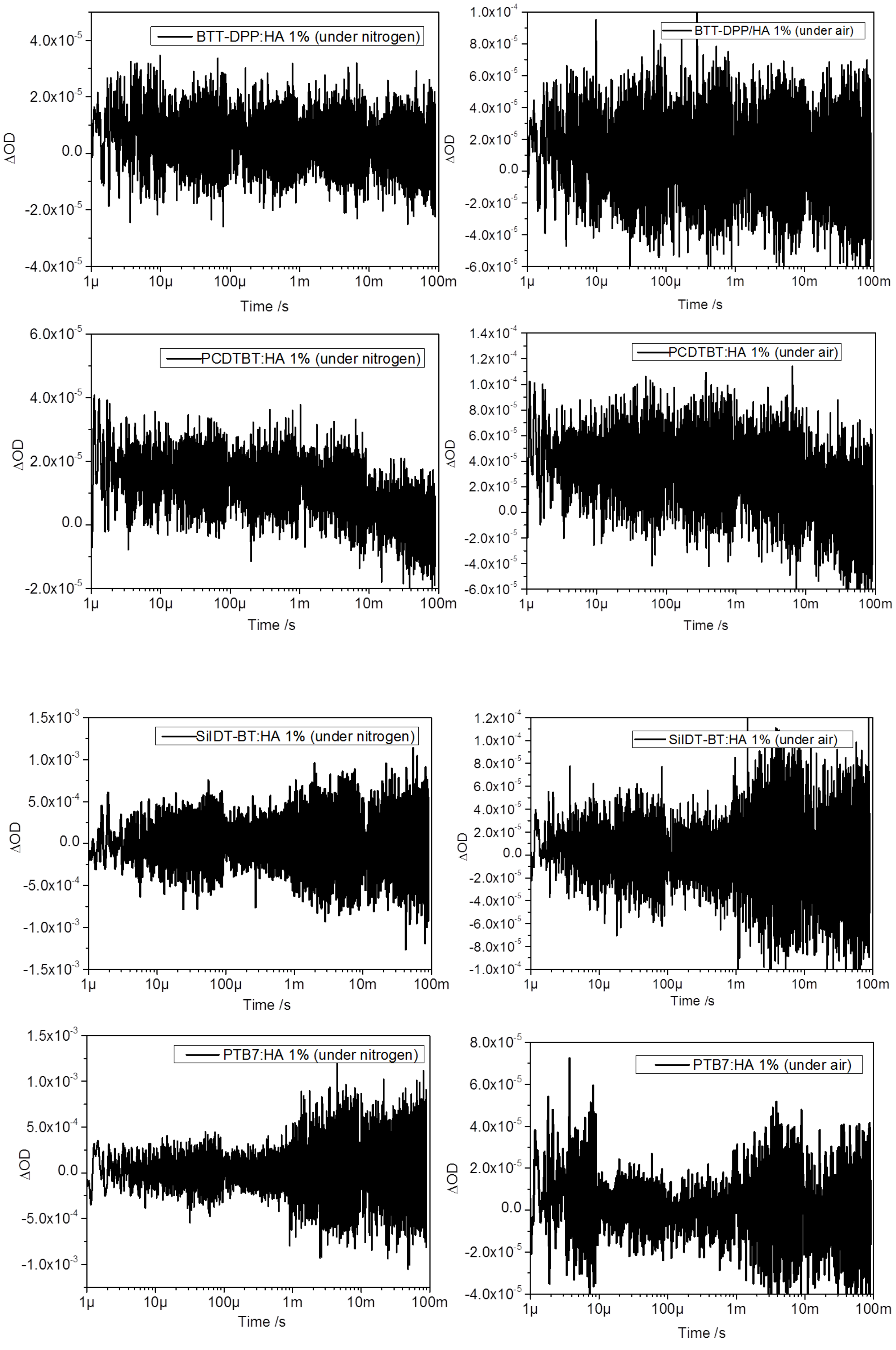

BTT-DPP:HA 1% (under nitrogen)
BTT-DPP/HA 1% (under air)
PCDTBT:HA 1% (under nitrogen)
PCDTBT:HA 1% (under air)
SiIDT-BT:HA 1% (under nitrogen)
SiIDT-BT:HA 1% (under air)
PTB7:HA 1% (under nitrogen)
PTB7:HA 1% (under air)
ΔOD
Time /s
1µ
10µ
100µ
1m
10m
100m

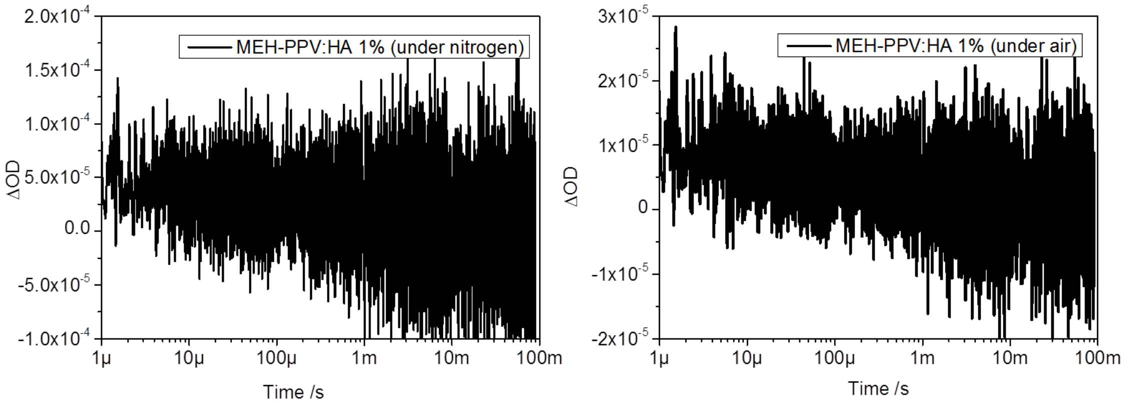

Figure S5c. Transient absorption kinetics both in a nitrogen and an air atmosphere at the polaron peak (in CdS blends) for pristine polymers with (1% wt./vol.) hexylamine processing additive in the film-forming solution. Excitation was at 567 nm at an energy of 21 ± 2 μJcm^{-2}. The probe wavelengths were 950 nm (SiIDT-BT), 1160 nm (BTT-DPP), 1200 nm (PTB7), 980 nm (PCDTBT), and 940 nm (MEH-PPV). No appreciable number of charges are observed in any of the pristine films.

Figure S6

Transmission electron microscopy

Transmission electron microscopy images were obtained using a JEOL 2000FX electron microscope operated at 200 kV. Samples for TEM were fabricated on water soluble sacrificial substrates and floated onto the surface of deionised water before being caught on 300 mesh copper grids for top-down imaging.

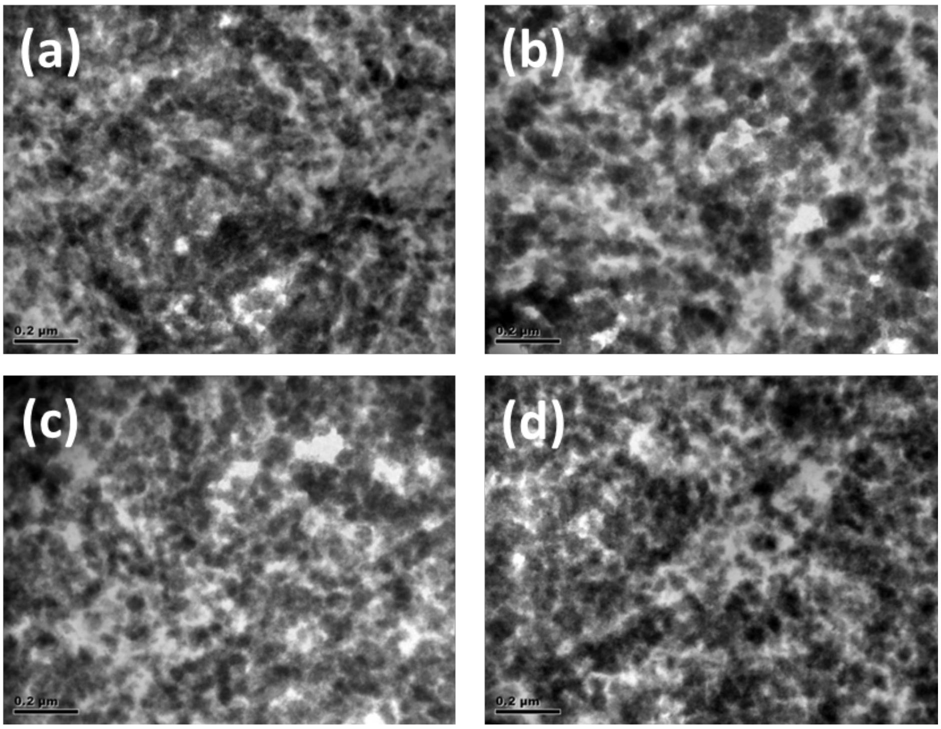

Figure S6a. Transmission electron micrographs of CdS:P3HT films with different ratios of hexylamine: (a) 0%, (b) 0.1%, (c) 0.5%, (d) 1%. The dark regions show the presence of CdS. Scale bars are 200 nm.

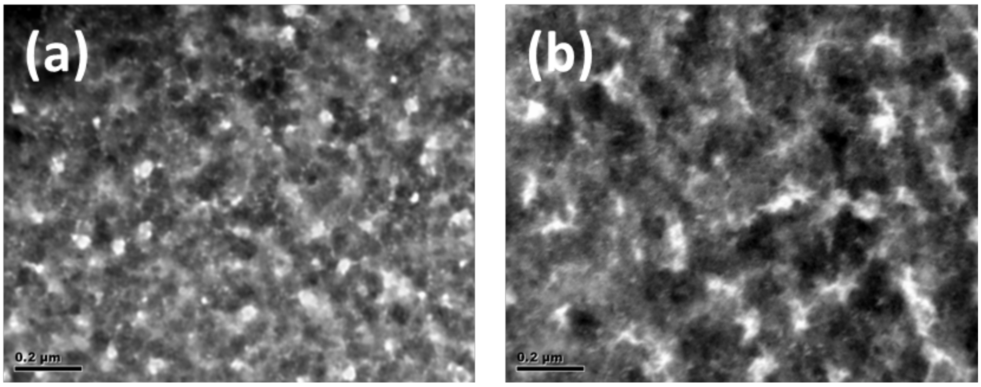

Figure S6b. Transmission electron micrographs of CdS:IF-DTBT films with different ratios of hexylamine: (a) 0%, (b) 1%. The dark regions show the presence of CdS. Scale bars are 200 nm.

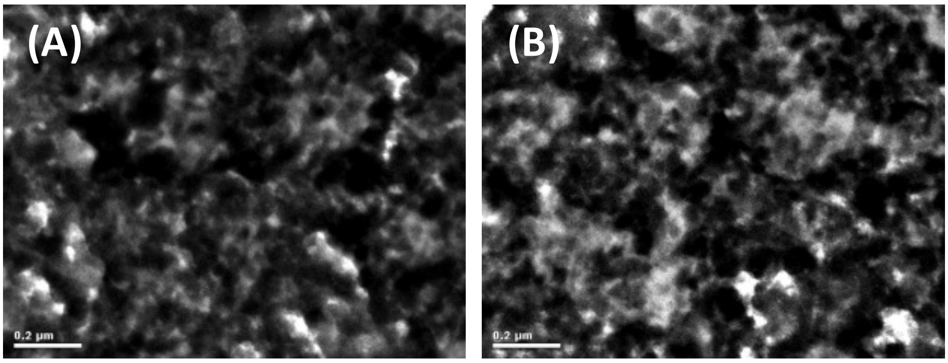

Figure S6c. Transmission electron micrographs of CdS:PCDTBT films with different concentrations of hexylamine: (a) 0%, (b) 1%. The dark regions show the presence of CdS. Scale bars are 200 nm.

<u>Figure S7</u>

<u>Polymer details</u>

The polymer P3HT was obtained from Merck and used without further purification. MEH-PPV was obtained from Sigma-Aldrich and used without further purification. The polymers PCDTBT and PTB7 were obtained from 1-Material and used without further purification. BTT-DPP, IF-DTBT and SiIDT-BT were synthesised as previously reported in Chem. Mater. **2011** *23* 768-770 and J. Phys. Chem. Lett. **2012** *3* 140-144.

<u>Figure S8</u>

<u>Electroluminescence intensity without normalisation</u>

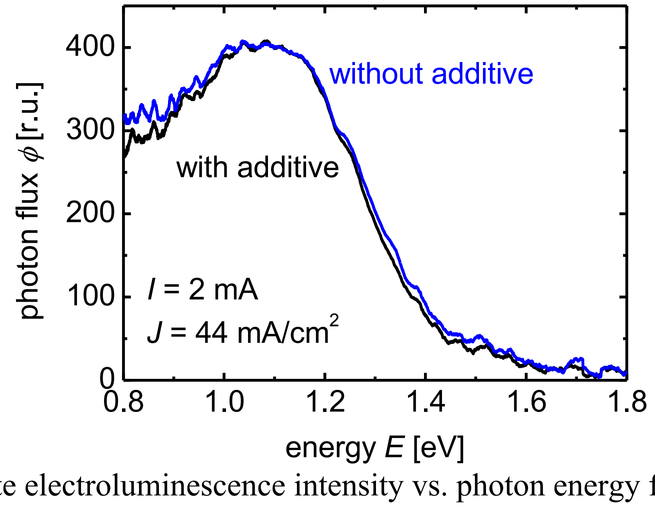

Figure S8: Absolute electroluminescence intensity vs. photon energy for the same P3HT:CdS solar cell shown in Fig. 4a in the main paper but not normalized to the peak value.
